# PLANNING FOR FUTURE CARE NEEDS: THE IMPORTANCE OF PERCEIVED NEED

**DOI:** 10.1093/geroni/igz038.1707

**Published:** 2019-11-08

**Authors:** Julie A Gorenko, Calandra Speirs, Candace Konnert, Claire McGuinness, Camille Mori

**Affiliations:** University of Calgary, Calgary, Alberta, Canada

## Abstract

Despite the demonstrated need to plan for future care needs, many individuals fail to engage in planning, often with negative consequences for their future health and well-being (Lee, Mason, & Cotlear, 2010). Theoretically, the propensity to utilize planning resources may be related to the perceived need for care in the future, a demonstrated predictor of the utilization of health and mental health services (Andersen, 1995; Karlin, Duffy, & Greaves, 2008). The purpose of this study was to examine perceptions of need for future care in combination with predisposing (age, financial security, attitudes towards planning) and enabling (anticipated support, satisfaction with family discussions about future care needs) variables in predicting planning behavior. The sample was comprised of 385 adults, aged 50 years and older (M=66.5, SD=9.3, range=50-92). Hierarchical regression analyses entered two well-established predictors, age and financial security in step 1, and attitudes towards planning, anticipated support, satisfaction with family discussions, and perception of need in step 2. Age and financial security explained 17% of the variance in planning; the addition of step 2 variables explained 33% of the variance and R-squared was significant (p<.001). All predictors were significant at p<.001, with the exception of anticipatory support (p<.05). These results support both the individual (i.e. positive attitudes, perceived need) and contextual nature of planning, in particular the belief that support will be available when you need it and the benefits of family discussions in facilitating planning. Recommendations for enhancing successful planning among individuals and their families will be presented.

